# The new field of ‘precision psychiatry’

**DOI:** 10.1186/s12916-017-0849-x

**Published:** 2017-04-13

**Authors:** Brisa S. Fernandes, Leanne M. Williams, Johann Steiner, Marion Leboyer, André F. Carvalho, Michael Berk

**Affiliations:** 1 IMPACT Strategic Research Centre, School of Medicine, Faculty of Health, Deakin University, Waurn Ponds, VIC 3216 Australia; 2 Barwon Health University Hospital, Geelong, Australia; 3 Laboratory of Calcium Binding Proteins in the Central Nervous System, Department of Biochemistry, Federal University of Rio Grande do Sul, Porto Alegre, Brazil; 4 Department of Psychiatry and Behavioral Sciences, Stanford University School of Medicine, Stanford, USA; 5 MIRECC, VA Palo Alto Health Care System, Palo Alto, CA USA; 6 Department of Psychiatry, University of Magdeburg, Magdeburg, Germany; 7 Department of Psychiatry, University Paris Est Créteil, AP-HP, INSERM U955, Translational Psychiatry, Fondation FondaMental, Créteil, France; 8 Translational Psychiatry Research Group, Department of Clinical Medicine, Faculty of Medicine, Federal University of Ceará, Fortaleza, Brazil; 9 Florey Institute for Neuroscience and Mental Health, Department of Psychiatry, University of Melbourne, Melbourne, Australia; 10 Orygen, The National Centre of Excellence in Youth Mental Health, University of Melbourne, Melbourne, Australia

**Keywords:** Precision medicine, Personalised medicine, Precision psychiatry, Systems biology, Biomarkers, Big data, Research domain criteria, Omics

## Abstract

**Background:**

Precision medicine is a new and important topic in psychiatry. Psychiatry has not yet benefited from the advanced diagnostic and therapeutic technologies that form an integral part of other clinical specialties. Thus, the vision of precision medicine as applied to psychiatry – ‘precision psychiatry’ – promises to be even more transformative than in other fields of medicine, which have already lessened the translational gap.

**Discussion:**

Herein, we describe ‘precision psychiatry’ and how its several implications promise to transform the psychiatric landscape. We pay particular attention to biomarkers and to how the development of new technologies now makes their discovery possible and timely. The adoption of the term ‘precision psychiatry’ will help propel the field, since the current term ‘precision medicine’, as applied to psychiatry, is impractical and does not appropriately distinguish the field. Naming the field ‘precision psychiatry’ will help establish a stronger, unique identity to what promises to be the most important area in psychiatry in years to come.

**Conclusion:**

In summary, we provide a wide-angle lens overview of what this new field is, suggest how to propel the field forward, and provide a vision of the near future, with ‘precision psychiatry’ representing a paradigm shift that promises to change the landscape of how psychiatry is currently conceived.

## Background

Precision medicine is “*an emerging approach for treatment and prevention that takes into account each person’s variability in genes, environment, and lifestyle*” [1]. Incorporating precision medicine to clinical practice is a priority that is gaining momentum. The ‘Precision Medicine Initiative’ [2], launched by President Obama in 2015, highlights its timeliness and relevance. This initiative aims to bring medicine into a new era by changing our concepts of how medicine is traditionally understood and applied in all clinical areas [3]. Psychiatry is a specialty that has not yet benefited from the advanced diagnostic and therapeutic technologies that now form an integral part of other clinical specialties, and thus the vision of precision medicine, as applied to psychiatry, namely ‘precision psychiatry’, promises to be even more transformative than in other fields of medicine that have already lessened the translational gap [4].

## How new is precision psychiatry? Is it really going to reshape psychiatry?

The ideas behind precision medicine are not new. At least to some extent, medicine has been personalised since its nascence. In 400 BC, Hippocrates established the scientific practice of medicine in Greece, and initiated the diagnosis and treatment of individuals according to four humours – blood, phlegm, and black and yellow bile [5]. In the 19th Century, the physiologist Claude Bernard, in his *Introduction to Experimental Medicine*, stated that, “*A physician is by no means a physician to living beings in general, not even physician to the human race, but rather, physician to a human individual, and still more physician to an individual in certain morbid conditions peculiar to himself and forming what is called his idiosyncrasy*” [6]. The intellectual father of precision medicine is considered to be Archibald Garrod [7], who published, in 1902, a paper entitled *The Incidence of Alkaptonuria: A Study in Chemical Individuality* [8]*.* In this paper, he wrote about the importance of individual ‘chemical differences’ in disease context: “*…the thought naturally presents itself that these* [alkaptonuria, albinism and cystinuria] *are merely extreme examples of variations of chemical behaviour which are probably everywhere present in minor degrees and that just as no two individuals of a species are absolutely identical in bodily structure neither are their chemical processes carried out on exactly the same lines*.” He also wrote a passage that has been taken as the first clear statement of the goals of precision medicine [7]: “*The task of the practitioner is far more than to apply the knowledge supplied to him from the laboratories; he … calls upon his experience to guide him as to how he may best help the particular patient* [manage his disease] *with the least possible damage*” [8].

While medicine has always had a personalised approach, it is not completely precise, or at least, not precise enough. The results achieved with instruments and information that physicians conventionally use, covered mostly by medical history and physical examination, are wanting. In this sense, precision medicine, as has been currently conceptualised and empowered by new available and powerful technologies, promises to finally fulfil its long-awaited ideal. If precision psychiatry lives up to its promises, it will not just be quantitatively ‘more precise’ than contemporaneous psychiatry, it will also be qualitatively different – and, in this case, a new field would emerge. An analogy could be made with arterial blood pressure. Blood pressure is measured in a continuous scale; however, an individual with a systolic blood pressure of zero cannot be considered in the same category as an individual with a systolic blood pressure of 300. An individual with a systolic blood pressure of zero is facing death in overt shock and requires treatment with drugs that increase blood pressure. On the other hand, an individual with a systolic blood pressure of 300 is in overt hypertensive crisis, and requires drugs that decrease blood pressure. Although both situations are manifestations of underlying alterations in the same continuous variable, i.e. blood pressure, they are so distinctly different that they are in different categories. The same applies to current psychiatry and precision psychiatry, when considering ‘preciseness’ dimensionally in a continuous scale. Indeed, this is even truer in psychiatry than in other clinical specialties; while it is the rule and not the exception to heavily rely on modern technology to inform clinical decisions such as diagnosis and treatment in other fields of medicine, in psychiatry, this is not the reality. The mere thought that this could be the case was non-existent or dismissed until recently. Thus, the introduction of relatively simple technologies as performed in other specialties, such as laboratory tests capable of guiding clinical diagnosis, will permanently change psychiatry. The term ‘paradigm shift’ was coined by the science philosopher Thomas Kuhn in his seminal work *The Structure of Scientific Revolutions* [9]. According to Kuhn, a paradigm is the entire worldview in which a current theory exists, and all of the implications that come from that worldview. This is based on features of the landscape of knowledge that scientists can identify around them. A paradigm shift is a fundamental change in the basic concepts and experimental practices of a scientific discipline. By Kuhn’s parameters, the mere idea that precision psychiatry is attainable is in itself a paradigm shift and, if precision psychiatry comes to be, this will represent an epistemological change in the field of psychiatry.

## The problem and the unique opportunity

The magnitude of the preeminent public health burden of psychiatry is, at least to some extent, a reflex of the poor knowledge that we possess about the pathophysiology of these disorders. This is further complicated as symptoms overlap considerably among different diagnoses whilst varying greatly among patients with the same diagnosis [10–13]. The endeavour of bringing precision medicine to psychiatry rests on, and simultaneously contributes to, the evolving knowledge of the biological pathways involved in the major mental illnesses. It builds on the aims of the Research Domain Criteria by the National Institute of Mental Health [14], which has initiated a research approach to generating a neurobiologically valid framework for classifying mental illness and for generating novel interventions related to neurobiological underpinnings.

**Fig. 1:**
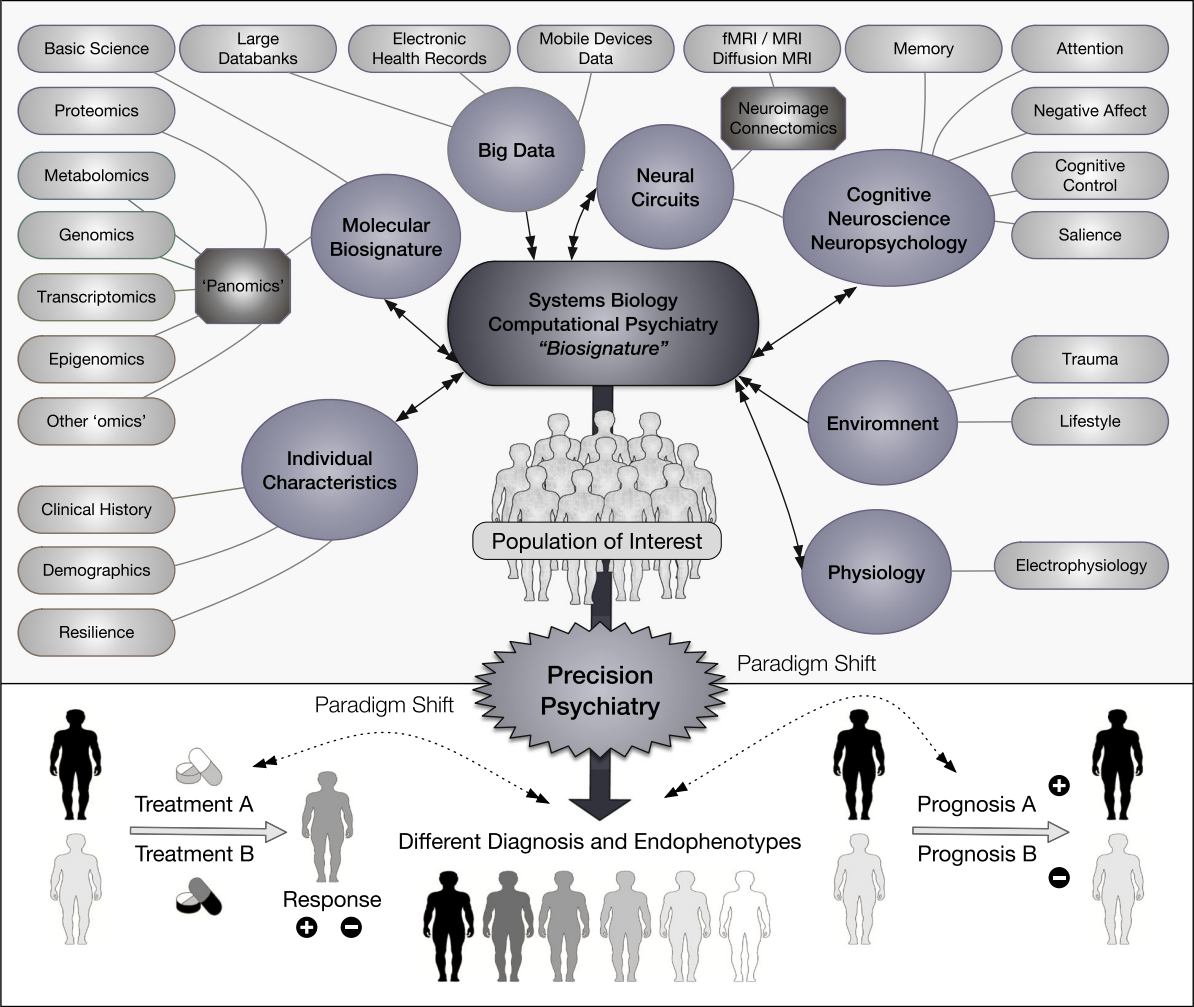
Domains related to ‘precision psychiatry’. Diverse approaches and techniques, such as ‘omics’, neuroimaging, cognition and clinical characteristics, converge to several domains. These domains can be analysed using systems biology and computational psychiatry tools to produce a biosignature – a set of biomarkers – that, when applied to individuals and populations, will produce better diagnosis, endophenotypes (measurable components unseen by the unaided eye along the pathway between disease and distal genotype), classifications and prognosis, as well as tailored interventions for better outcomes. The bottom-up approach from specific areas (such as metabolomics) to domains (such as molecular biosignature), to systems biology and computational psychiatry, to a resultant biosignature, can also be reverted to a top-down approach, with specific biosignatures being analysed to better understand domains and its specific components. Components and domains are not mutually exclusive, and a subject can belong to more than one component or domain; for instance, ‘large databanks’ can belong to data from ‘neuroimaging’, ‘mobile devices’ and ‘panomics’, all of which are put as different domains. After the establishment of precision psychiatry, persons considered to belong to the same group (agglomerate of persons in grey) will be reclassified into different diagnosis and endophenotypes. Further, after accomplishing precision psychiatry, it will be possible to more accurately predict response or non-response to treatment, as well as better prognosis

What was once considered an elusive task, since it is unreasonable to expect that conditions with such clinical and pathophysiological diversity might have a simply definable pathological mechanism, is now much more approachable. This enormous challenge in brain research needs to be explored at multiple units of analysis within the biological system, including data from physiological recordings, brain imaging, ‘omics’ biomarkers, environmental exposures and self-reported experience. These units of data also need to be combined with ecological momentary assessments that track real-time changes in daily function. All these methods entail accumulations of massive datasets that require new analytic approaches for interpretation. These new approaches will rely on developing models that integrate across scales (from micro to macroscales) across time, and that harness the perspectives from interdisciplinary work among mathematicians, physicists, biologists and clinicians in order to achieve an appropriately integrative understanding of mental illness as disorders of the brain (Fig. 1) [15]. Mental disorders have not always been considered ‘brain disorders’ or ‘brain diseases’ [16]; rather, the term ‘brain disease’ has been more consistently used to refer to neurological conditions associated with a discrete lesion or degenerative process. This usage may reflect our limited understanding of the real-time coordination of the brain, and the fact that psychiatric disorders are functional expressions of subtler pathologies. Given the advent of brain imaging techniques with sufficient spatial and temporal resolution to quantify neural connections in vivo, it is the right time to reformulate our understanding of mental illness as disorders of brain functioning [17].

This is a very ambitious endeavour. However, the timing for successfully carrying out this task has never been more propitious. The odds of turning this ideal into reality have considerably increased with the development of powerful biological tools, methods, brain imaging and physiological techniques, as well as assessment of behaviours and life experiences, to characterise patients along with the advanced computational tools capable of analysing large datasets. The availability of massive information – ‘big data’ – provided by the acquisition of biological data on scale and by incorporating data from electronic devices such as smartphones is unprecedented, and is one of the multiple factors that now allows the analysis of diverse patient characteristics to be considered [18]*.*

## The convergence of different fields

An interdisciplinary approach offers the potential to identify ‘biomarkers’ – “*a defined characteristic that is measured as an indicator of normal biological processes, pathogenic processes, or responses to an exposure or intervention, including therapeutic interventions*” [19] – as indicators of pathophysiology, risk for pathophysiology, or treatment outcome that can be measured by gene and/or brain assays or their combination with environmental factors. Considering that no single biomarker will probably define any psychiatric disorder as defined by traditional diagnostic boundaries [13, 20, 21], it will be essential to pursue in parallel theory- and data-driven discovery approaches to delineate the multivariate and combinatorial profiles of biomarkers (across units of analysis) that account for the heterogeneity of mental illnesses as they manifest clinically.

Arguably, the most logical way of obtaining hypothesis-free and data-driven approaches in the neurobiology of psychiatric disorders is making use of ‘omics’ techniques. Genomics, epigenomics, transcriptomics, proteomics, metabolomics, metagenomics and lipidomics are capable of independently providing valuable information about the neurobiology of these psychiatric conditions. Further, when combined with a multi-omics approach, in what is called panomics, and analysed using system biology computations, they might unveil the underlying biological pathways involved in psychiatric disorders.

Implications of systems understandings include the principles of redundancy, whereby multiple elements with overlapping functions are present for backup, of modularity, such that failure of one element does not lead to system-wide failure, and of structural stability, in which intrinsic mechanisms exist to enhance systemic stability. This implies that network-wide omics approaches are needed to capture the interactivity of these multiple overlapping elements [22]. This neurobiology information can be aggregated to the now abundant behavioural data available, including thorough mobile technology [23]. Psychiatry is concerned with the diagnosis, causes and treatment of mental disorders. The strategies delineated here, when employed conjointly, have the possibility to lead to the discovery of systems biomarkers capable of aiding clinicians in the diagnosis, prognosis, prediction of response to treatment and treatment choice, while also providing clues to the molecular basis for the development of new and more tailored treatments (Fig. 1). Given the complexity of psychiatric disorders, it may be anticipated that the precision psychiatry paradigm may not lead to the development of entirely new molecular compounds in the next few years, which would require new mechanisms of action for treatments based on a better understanding of the mechanisms underlying the discrete disease processes as opposed to syndromic diagnoses. A more likely expectation for the next years is that the precision psychiatry paradigm will lead to the discovery of biomarkers able to guide treatment choice and predict treatment response to commonly used drugs such as antidepressants and antipsychotics. Indeed, this is already occurring. Just to cite a few examples, C-reactive protein has been shown as a predictor of differential response to escitalopram or nortriptyline [24], and both brain magnetic resonance imaging and childhood trauma have been associated with poor response to antidepressants [25, 26].

## To be precise: what does the ‘precision’ in precision psychiatry refer to?

The noun ‘precision’ in the term precision psychiatry is, grammatically, a modifier to the word ‘psychiatry’. Its dictionary definition is “*the quality, condition, or fact of being exact and accurate*” and “*refinement in a measurement, calculation, or specification*” [27]. Thus, according to this lexical definition, ‘precision’ in precision psychiatry conveys that the latter has a foundation in measurement. The original term, personalised medicine, was changed to precision medicine to emphasise that its technologies and treatments are not developed for each individual patient, as the term personalised suggests, but rather that a high level of exactness in measurement will be achieved such that, eventually, it will be personalised. It can be conceptualised as a highly sophisticated and intricate classification system, where infinitesimal categories will, ideally, attain perfection in a detailed multidimensional classification. Again, the application of these elaborated patterns to individuals will eventually lead to a personalised treatment, but will differ from personalised medicine. In precision psychiatry, for instance, a given patient would receive an existent treatment pre-established according to the patient’s disease class, and not a medication that would be specifically created for that individual following consideration of their unique features, as would be the case in ‘personalised psychiatry’.

An analogy can be made using cardiology, a specialty far more advanced than psychiatry; 70 years ago tools to measure the un-observable aspects of heart structure and function were not available, now, these are taken for granted. Physicians would have endeavoured to personalise their assessments to each patient, yet they were not able to observe the heart’s behaviour or how it related to observable symptoms prior to the incorporation of measurements. The Framingham study, for example, spawned the assessment of standard vitals, along with a subsequent range of imaging techniques to determine whether the underlying biological problem is one of function (requiring perhaps a pacemaker or medication) or one of structure (requiring perhaps surgery). It would be routine to take serial images to provide a baseline from which to assess each individuals’ recovery and their subsequent risk for another acute problem. These measurements would also inform the importance of a personalised plan that considers the whole individual, from observables to non-observables, including lifestyle, diet and other techniques. The major challenge that precision psychiatry faces is that psychiatry does not yet use measurement to track the equivalent of vitals and images of the organs of interest, namely the brain and alterations in peripheral measures such as blood.

## Envisioning new landscapes: where to go from now?

A maxim of management is that “*the best way to predict the future is to create it*” [28]. By having a vision of how the future should be, we can begin to plan. However, the mere conception of a vision is not enough – working towards it is essential. As a small step in animating the vision of the field of precise medicine as applied to psychiatry, adoption of the term ‘precision psychiatry’ – first coined by Vieta et al. [4] – will assist in creating a stronger identity. Consequently, it will reinforce commitment to a field that is a reflex of the unfolding of a paradigm shift in which neuroscience will be integrated into our models of psychiatry. This is what was performed in oncology, with ‘precision oncology’ being now one of the most developed areas of precision medicine [29]. Although the ‘War on Cancer’ started in 1971, and is only now providing dividends, it is reasonable to expect that the developments in psychiatry will be faster, since we now possess technologies that were not available in the last millennium. Another advantage that psychiatry has is not being the first. Psychiatry can learn from the past successes and failures of oncology and almost all other clinical specialties. Additionally, psychiatry will need considerably more modest changes than oncology did 40 years ago to debut in the precision medicine paradigm.

The development of the field of precision psychiatry in its full totality is a gigantic but addressable enterprise that will most likely require synergy between academia, industry and government [30]. Each of these three spheres possesses unique but complementary skills [31]. In academia, science is defined and practiced according to the traditional Aristotelian view. Scientists, including neuroscientists, mostly describe and explain nature through observation and experiment – understanding nature is an end in itself. To aid precision psychiatry fully coming to life, the marriage of science and technology is paramount, since the development of precision psychiatry involves, not only unveiling nature, but also creating new technologies, requiring applied, inventive technological efforts.

An entrepreneurial disposition is also needed for commercialisation of the developed technologies. This is a task suitable for a partnership of scientists with industry [32]. Further, government has the role of creating new policies and providing strategic direction, as done by the National Institute of Mental Health with the launching of the Research Domain Criteria and of the Precision Medicine Initiative. In addition, government is best suited to regulate commercial medical devices and new pharmacological compounds. In the United States, this is within the scope of the Food and Drug Administration and regulated according to the Clinical Laboratory Improvement Amendments to assure that the new tests developed possess both analytical and clinical validity [33].

Finally, all of the above steps will mean nothing if they are not applied. To apply precision psychiatry, clinical scientists will have to develop clinical guidelines specifying how the new developed technologies should be employed and clinically evaluated. This last step is crucial; since psychiatrists are not used to relying on instruments such as medical tests, the introduction of clinical guidelines, clearly informing how such new technologies should be employed and evaluated in clinical practice, will be necessary to guarantee the last translational step. Only when precision psychiatry is realised in ‘real life’ will it keep its promises [34–36].

## Conclusions

The ultimate goal of precision medicine as applied to psychiatry – ‘precision psychiatry’ – is to seek better lives for those suffering from mental illness. This will only be accomplished with the development of tools capable of providing better and more accurate diagnosis, of ascertaining prognosis, guiding treatment and predicting response to treatment, and aiding the development of new and better pharmacological and non-pharmacological treatments. If achieved and applied, ‘precision psychiatry’ will be of great consequence and will redesign the current landscape of mental illness.

Paraphrasing Claude Bernard, whose goal in life was to establish the use of the scientific method in medicine, “*Art is I; Science is We*”. Hopefully, the day will come when science is fully incorporated into psychiatry – a medical specialty deemed as highly subjective; then, we will collectively change from ‘I do’ to ‘We do’ and we will be practicing precision psychiatry. 

*Annotations:* Rectangles indicate specific components; circles indicate domains; agglomerate of persons in grey indicates different individuals grouped together that, after precision psychiatry, will be better recognised. Bi-directional arrows, bi-directional relationships; uni-directional arrows, uni-directional relationships.
